# Recent Advances in Micro-Electro-Mechanical Devices for Controlled Drug Release Applications

**DOI:** 10.3389/fbioe.2020.00827

**Published:** 2020-07-29

**Authors:** Luis Abelardo Villarruel Mendoza, Natalia Antonela Scilletta, Martin Gonzalo Bellino, Martin Federico Desimone, Paolo Nicolas Catalano

**Affiliations:** ^1^Departamento de Micro y Nanotecnologia, Instituto de Nanociencia y Nanotecnología, CNEA-CONICET, San Martín, Argentina; ^2^Instituto de Nanociencia y Nanotecnología, CNEA-CONICET, San Martín, Argentina; ^3^Universidad de Buenos Aires, Consejo Nacional de Investigaciones Científicas y Técnicas (CONICET), Instituto de la Química y Metabolismo del Fármaco (IQUIMEFA), Facultad de Farmacia y Bioquímica, Buenos Aires, Argentina; ^4^Universidad de Buenos Aires, Facultad de Farmacia y Bioquímica, Buenos Aires, Argentina

**Keywords:** micro-electro-mechanical systems, drug delivery, microfabrication, implantable MEMS devices, actuation mechanisms, transdermal devices

## Abstract

In recent years, controlled release of drugs has posed numerous challenges with the aim of optimizing parameters such as the release of the suitable quantity of drugs in the right site at the right time with the least invasiveness and the greatest possible automation. Some of the factors that challenge conventional drug release include long-term treatments, narrow therapeutic windows, complex dosing schedules, combined therapies, individual dosing regimens, and labile active substance administration. In this sense, the emergence of micro-devices that combine mechanical and electrical components, so called micro-electro-mechanical systems (MEMS) can offer solutions to these drawbacks. These devices can be fabricated using biocompatible materials, with great uniformity and reproducibility, similar to integrated circuits. They can be aseptically manufactured and hermetically sealed, while having mobile components that enable physical or analytical functions together with electrical components. In this review we present recent advances in the generation of MEMS drug delivery devices, in which various micro and nanometric structures such as contacts, connections, channels, reservoirs, pumps, valves, needles, and/or membranes can be included in their design and manufacture. Implantable single and multiple reservoir-based and transdermal-based MEMS devices are discussed in terms of fundamental mechanisms, fabrication, performance, and drug release applications.

## Introduction

The design and development of novel materials and devices for medicine is one of the exciting new areas of bioengineering and biotechnology, which is rapidly gaining the attention of the scientific community (Zhang et al., 2011; Hardy et al., 2013; Copes et al., 2019; Hu and de Vos, 2019; Tamay et al., 2019). Biofabrication is providing scientists and clinicians the ability to produce engineered devices with desired shapes, chemical composition, and biological functions (Echave et al., 2019; Scilletta et al., 2019; Debons et al., 2020; Frayssinet et al., 2020). In the last few decades, several biomaterials and nanomaterials have been proposed and develop to this end (Desimone et al., 2010; Scodeller et al., 2013; Alvarez et al., 2014; Mebert et al., 2017). Indeed, the design and optimization of new drug delivery systems that may help to limit multiple application doses and improve patient compliance is advancing rapidly (Boccardi et al., 2015; Hardy et al., 2016; Mebert et al., 2016). Biotechnology and bioengineering are rapidly moving toward the development of personalized tools to achieve more predictable and optimal treatments (Orive et al., 2020).

Micro-electro-mechanical systems (MEMS) are technological devices which can be well-defined as miniaturized mechanical and electro-mechanical elements creating miniature integrated systems or devices (Lyshevski, 2002; Villanueva et al., 2016). They varied from comparatively simple structures having no moving parts, to very complex systems with numerous moving elements under the control of combined microelectronics. The interdisciplinary of MEMS nature exploits from the integration of a diverse range of technical areas including integrated circuit techniques, chemistry and chemical engineering, mechanical engineering, materials science, electrical engineering, as well as microfluidics, biomedical engineering, and optics (Judy, 2001). MEMS production technologies uses high performance processing that usually includes addition or subtraction of 2D layers on a substrate based on mainly photolithography techniques and a variety of thin-film deposit and patterning technologies, together with selective chemical etching (Madou, 2002).

Current MEMS devices have the capability to sense, actuate and control on the micro-scale, and produce effects on the macro-scale. MEMS have been recognized as one of the most auspicious technologies of the 21st Century and have the future to revolutionize both consumer products and industry. MEMS are considered the second micro-manufacturing revolution after the first revolution based on semiconductor microfabrication. These microsystem-based devices have the prospective to dramatically get better all our lives. MEMS can be found in devices ranging across electronic, communication, and medical applications (Ho and Tai, 1998; Gardner and Varadan, 2001; Rebeiz, 2004; Mohammadi Aria et al., 2019). Current MEMS include for example: inkjet printer heads, accelerometers, computer disk heads, display chips, optical switches, blood pressure sensors, biosensors, microvalves, and numerous other products that are manufactured in great commercial volumes (Kaajakari, 2009).

The experience gained from the early MEMS applications has tailored their emerging technology for biotechnological developments including water and environmental monitoring, DNA sequencing and drug discovery (Bashir, 2004). Novel drug delivery methods to release a specific dose of a drug with a certain rate, during an adequate period of time in the targeted tissue and facilitating patient self-administration are becoming increasingly necessary (Orive et al., 2003; Geipel et al., 2007; Ruggiero et al., 2018; Singh et al., 2019; Tharkar et al., 2019; Chen Y. et al., 2020; Deng et al., 2020; Hwang et al., 2020). In this sense, advanced biomedical technology will comprise implantable MEMS devices of proven biocompatibility (Voskerician et al., 2003), to carefully release drugs into the body from micro-chambers hosted in the device, eliminating the need for injections or needles (Grayson et al., 2004; Vasudev and Bhansali, 2013). The delivery of antibiotics, anti-inflammatories and analgesics is one such promising application, as also is the delivery of chemotherapy drugs or even hormones as the archetypical insulin (Shawgo et al., 2002; Chen et al., 2018). It is to note that these investigations could be enhanced by the incorporation of stimuli sensitive and/or targeted nanocarriers (Couvreur, 2013; Gonzalo et al., 2013; Jhaveri and Torchilin, 2014; das Neves et al., 2015; Kumar et al., 2018) within MEMS devices and the use of microfluidic organ-on-a-chip platforms for testing these drug delivery systems (Bhise et al., 2014). An envisioned generation of MEMS chips could also interact with MEMS and hydrogel-based sensors embedded in the body itself to respond to own internal signals (Caldorera-Moore and Peppas, 2009; Peppas and Van Blarcom, 2016).

This review introduces the field of micro-electro-mechanical devices for controlled drug release applications and is organized as follows. A comprehensive implantable MEMS devices description from single to multiple reservoirs is presented together with their actuation mechanisms and current applications and challenges. Transdermal delivery devices are particularly described in the subsequent section. Finally, some concluding remarks and perspectives are outlined.

## Implantable MEMS Drug Delivery Devices

Drug delivery implantable devices are characterized by containing reservoirs loaded with drugs to be released, which are the most critical parts of them. Reservoir material constitution must not only be biocompatible on the outside but also inert on the inside, as this part is directly in contact with the drugs to be delivered (Grayson et al., 2004; Lee et al., 2018). Hence, polydimethylsiloxane (PDMS), polyacrylamide (PAA), medical grade silicone rubber and Pyrex© are the most widespread materials suitable for reservoir construction. They have well known physicochemical properties that direct new developments toward their utilization. Their biocompatibility, bonding and optical transparency are some of the desirable characteristics found in these kinds of materials (Uvarov et al., 2016a). Recent microfabrication processes offer a lot of advances in size terms to achieve admissible reservoirs (Randall et al., 2007). In this sense, reservoir size should be big enough to load the appropriate drug doses and small enough to ensure that actuation mechanisms are effective in the release of drugs and that the total size of the device is not significantly increased, considering their implantable destination. Nowadays, miniaturization of implantable devices is truly of great relevance (Staples et al., 2006). As, on one hand, devices first functionality tests are developed in small laboratory animals, and on the other hand, the final target is human tissue, the reduction of device size is always welcome. In order to accomplish drug delivery in exact doses and in a controllable manner, MEMS drug delivery devices are usually based on single or multiple reservoirs where drugs are loaded (Zhou et al., 2012).

### Single Reservoir-Based Devices

The most recent reports of MEMS devices for drug release applications have proposed to use a single reservoir to load different drug formulations. In these devices the total amount of drug contained in the reservoir is dosed by pumps using different actuation mechanisms to achieve an exact and controlled release of the drug. Different materials were employed for reservoir constitution, mostly depending on the actuation exerted (Zhang and Wei, 2016). In this section recent advances in single reservoir MEMS drug delivery devices are discussed based on their actuation mechanisms. A summary of them can be found on Table 1.

**TABLE 1:** Single reservoir-based MEMS devices for drug delivery applications.

Actuation mechanism	Device dimensions	Drugs tested	Main materials used	References
Piezoelectrical	(8 × 8) mm × 100 μm	Water based fluids	Silicon wafer Au PDMS	Rao et al., 2018
Piezoelectric	(700 × 130) μm	N/A	PZT-5H Quartz PDMS	Sateesh et al., 2018
Phase-change	(7 × 13 × 1) mm	N/A	Silicon TEOS Parylene-C Gallium Glass	Johnson and Borkholder, 2016
Phase-change	8 mm × 8 mm × 3 mm	Sodium salicylate	Parylene-C PDMS Microtubing Paraffin wax	Forouzandeh et al., 2019
Electrochemical	10 mm long, 10 mm wide, and 2 mm high	Fluorescent polystyrene microparticles	Cu PTFE PDMS Au electrodes Silicon substrate	Song et al., 2017
Electrochemical	20 × 15 × 8.1 mm, L × W × H	N/S	Parylene Pt Rigid glass substrate Nafion^®^	Cobo et al., 2016
Magnetic	5 mm × 3 mm × 12 mm and 2 mm × 1 mm × 12 mm	Methylene blue Docetaxel	PDMS Iron oxide particules	Zachkani et al., 2015
Electrochemical	10 mm × 10 mm × 2 mm	Adrenaline	PDMS PO Pt/Ti	Liu et al., 2015
Transdermal	21 mm Ø	N/A	PDMS Acrylic Silicon	Cantwell et al., 2014
Electrochemical	13 mm × 13 mm × 3.5 mm	Doxorubicin hydrochloride	PDMS Silicon wafers Ti/Au	Song et al., 2013
Electrochemical	N/S	N/S	Parylene Pt/Ti Soda lime wafer Nafion^®^	Sheybani et al., 2013a
Electrochemical	N/S	siRNA-gold nanoplex drug (HNB-001)	Soda lime wafer Parylene C Pt/Ti MDX4-4210 Silicone rubber	Gensler et al., 2012
Magnetic	(6 Ø × 0.59) mm	Methylene blue	PDMS Iron oxide nanoparticles	Pirmoradi et al., 2011a
Magnetic	(6 Ø × 0.59) mm	Docetaxel	PDMS Iron oxide nanoparticles	Pirmoradi et al., 2011b
Piezoelectric	(8 × 8) mm × 100 μm	Waterbased fluids	Silicon wafer Cr/Au PZT	Geipel et al., 2007

*N/S, not specified; N/A, not aplicable; Au, gold; Pt, platinum; Ti, titanium; Cr, chromium; Cu, copper; PDMS, polydimethylsiloxane; PO, polyolefin; PTFE, poly(tetrafluoroethylene); PZT/PZT-5H, piezoelectric materials; MDX4-4210, biomedical grade silicon elastomer; TEOS, tetraethyl orthosilicate.*

#### Micropump-Based Devices

Micropumps, essential components of microfluidic devices, were proposed to achieve the aim of releasing a specific dose of a drug with a certain rate and during an adequate period of time in a targeted tissue as they are responsible for generation of fluid movement (Kalra and Mashuq un, 2017). Small and compact pumps can be incorporated into delivery devices and precisely control the dosage to be delivered into the body. The micropumps can be programmed for instance to administer drugs at a constant rate throughout the day, thus eliminating any surges or deficits of the drug in patient’s bloodstream. Dosage and timing are critical to the study of drug effects on the body; hence, selecting an adequate pump is critical to optimal device function. Numerous developments have been reported in this way. Actually, a noticeable large number of designs allow the combination of uncountable type of arrangements to improve MEMS drug delivery system (Joshitha et al., 2017). Nowadays, pump size, low heat generation, precise metering capability, small power consumption, and the ability of system integration are critical parameters for life science applications (Cobo et al., 2015). Micropumps are usually classified into mechanical (displacement) and non-mechanical (dynamic). Piezoelectric, magnetic, and those based on materials phase change are among the most reported mechanical micropumps. On the other hand, the most relevant non-mechanical micropumps reported are those based on electrochemical induced forces to move fluid. All these types of micropumps can be designed to be miniaturized for implantation purposes. In addition to their sizes, the need of an external power source and energy required for operation become critical. These factors will determine the final size of the device and the feasibility for implantation.

##### Mechanical Micropumps

Mechanical micropumps use the movement of components such as oscillating diaphragms to pump a fluid by applying pressure (Mohith et al., 2019). These, also called displacement micropumps, consist of two main components for fluid pushing and halting and develop a pulsating flow due to their periodicity. Their general construction includes a flexible membrane or diaphragm, an actuator, a pumping chamber, an inlet, and an outlet. The oscillatory or reciprocal movement of the membrane is driven by a physical actuator that produces the pressure difference for fluid pumping. Piezoelectric, magnetic, and material phase change, are among the physical driving forces used in mechanical micropumps based MEMS devices for drug delivery applications.

###### Piezoelectric-based micropumps

In conventional piezoelectric micropumps, a middle membrane goes up and down being driven by the activation of an actuator. These membranes are commonly made of lead zirconate titanate (PZT), an artificial ceramic material with a strong piezoelectric effect. These kinds of pumps alternate suction and compression phases causing the flow entering and exiting the chamber through valves. Piezoelectric pumps are the most common devices made since 1990s. Smits (1990) originally described a three piezoelectric valve peristaltic pump. In this development the pump was constituted by a piezoelectric disk attached to a glass membrane, which was carried on a silicon wafer supported by a flat glass bottom plate. Glass membrane attaching to the silicon wafer was performed by anodic bonding and the device was built by etching techniques. At the beginning of any pumping cycle, the piezoelectric disk was electrically deflected in a convex shape. This deformation allowed sucking of the medium from the inlet into the generated cavity under the piezoelectric material, which was therefore connected with the backside channel and the inlet tube. The next piezoelectric membrane was convexly deflected in the same way as the previous one, allowing the medium to flow into the hollow under the second membrane. After that, closing of the first element was performed by decreasing its voltage to zero or negative, and the medium was gathered under the second element. Later, the third membrane was deflected, causing the channel on the back to be connected with the outlet. After second element voltage shut down, the stored fluid was pushed in the channel toward the final open element. Finally, by decreasing the voltage of this third element the medium is free to flow through the outlet pipe. At the end of the pumping cycle the three membranes were closed and a new one could start. With this design a high pumping rate of up to 100 μL min^–1^ was reported to be theoretically feasible.

Going forward to recent years, a theoretical design and simulation of a single piezoelectric micropump composed by zirconate titanate (PZT)-5H material, quartz and PDMS was reported by Sateesh et al. (2018). Although piezoelectric pumps offer fast response times and high forces, high voltage needs and special care must be taken into account when mounting PZT discs. The aim here was to reduce the power consumption by means of flow speed increase. Laminar and turbulent flows were considered in the analysis of device structural materials. The authors were especially interested in analyzing laminar flows resulting when pumping biological media. The analysis was made by changing the inlet, outlet, and channel dimensions. PDMS was chosen as the material to support the bending of the piezoelectric membrane owing to its transparency, high yield strength, good flexibility, inexpensiveness and biocompatible properties. This would be placed over two electrodes, ground and active. A quartz channel with inlet and outlet on PDMS membrane was considered. Application of voltage to the electrodes would cause the piezoelectric material to deform and vibrate, pumping the drug out of the channel. This study demonstrated that increases in channel and outlet widths resulted in lower flows. Although this design offers flexibility and controllability for drug delivery platforms (Khare and Singh, 2014), even reducing component dimensions, low pumping rates were achievable with either turbulent or laminar flow regimes when using this single piezoelectric element. A variation of this design in which the device is integrated with a micro-needle to deliver the pumped drug into the patient’s body was analyzed and simulated (Rao et al., 2018). Reliability, biocompatibility and required fabrication procedures were considered in the election of silicon as micro-needle material. Much higher flow rates were achievable here (up to 4.7 mL min^–1^) by analyzing changes in voltage and piezoelectric materials.

In another approach, a two-stage micropump, designed for metronomic drug delivery was reported (Geipel et al., 2007; Mahnama et al., 2014). This system had two back to back active valves with two separate piezo-actuators and worked in a three phase regimen, including: re-fill, transfer, and delivery phases. The device was fabricated in a two bulk silicon process which utilized standard lithography and included chromium/gold electrodes. The cavities and the fluidic channel ports were made by KOH etching, while the pump chamber and the valve lips were fabricated by deep reactive ion etching in separate wafers which were then bonded by low temperature silicon wafer bonding. This design stands out for its independence from the flow back pressure (which was tested in the range of 0.1–50 μL min^–1^), allowing a precise volumetric dosing of aqueous fluids.

**FIGURE 1:**
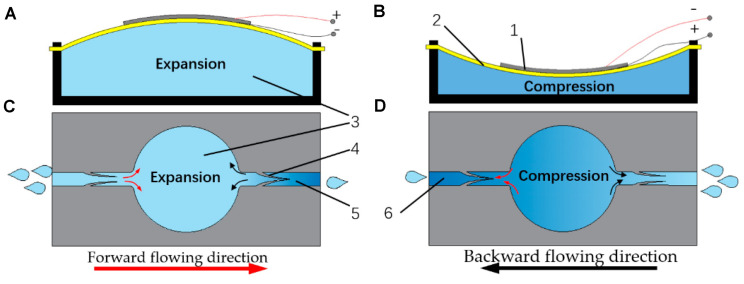
The working principle of the proposed lead zirconate titanate (PZT) pump: **(A)** the pump chamber in suction stroke; **(B)** the inhaling principle during suction stroke; **(C)** the pump chamber in compression stroke; **(D)** the exhausting principle during compression stroke; (1) the piezoelectric ceramic; (2) the brass substrate; (3) the pump chamber; (4) the compliant structure; (5) the outlet channel; (6) the inlet channel. Reproduced from Bao et al. (2019) with permission from MDPI Open Access Journals.

Recently, a millimeter-scale piezoelectric pump with built-in compliant structures was designed, fabricated, and experimentally tested by a group of researchers (Bao et al., 2019). This device had a chamber of 12 × 1.1 mm (diameter × height), a piezoelectric vibrator, and two pairs of valve featured elements on the flowing channel (Figure 1). PDMS, polyethylene, parylene, SU-8 photoresist, hydrogel, among others, were considered for device building. The manufacturing methods included computer numerical control (CNC) machining, 3D printing technology, and MEMS fabrication methods such as wet etching and deep reactive ion etching. The flow channel was 2 mm in width and 1.5 mm in height. The entire device was made in two parts: (1) the upper part that mainly included a piezoelectric vibrator mount, and two grooves at its ends; (2) the lower part that included a pump chamber, inlet and outlet channels, four grooves to mount the valve featured elements, and two grooves at the end of the flowing channels. Both parts were bonded by ultraviolet epoxy. Performance results showed that the flow rate positively correlated to the voltage and a maximum of 3.6 mL min^–1^ at 210 V and 80 Hz was achieved. The authors did not described the design of a micrometer sized pump but considered that the valve featured elements were not importantly limited by the size of the channel and that the developed piezoelectric pump will be able to be miniaturized by MEMS processing methods turning it a good candidate for microfluidic applications like drug delivery systems.

Another recent study analyzed the theoretical performance of a piezoelectric micropump for drug delivery applications (Farshchi Yazdi et al., 2019). The proposed micropump is based on silicon diaphragms actuated at 60 V and on flow input and output passive valves. The most important features of this design were that the device could be fabricated using two silicon wafers and that the use of an elliptical chamber could decrease dead zones and increase outflow. The performance and efficiency of the micropump were improved by optimizing geometrical features, which was enabled by the computational model. In this way, an outflow of 1.62 μL min^–1^ at 10 Hz was increased up to 2.5 μL min^–1^, paving the way for the development of micropump prototypes for biomedical applications following the proposed design.

Piezoelectric micropumps have shown to offer high actuation forces, fast response times and precise volumetric dosing of aqueous fluids. However, their manufacture is complex and special care must be taken in processing and mounting PZT discs. They also require a comparatively high actuation voltage. There are no reports of implantable MEMS devices based on piezoelectric pumps which were tested *in vivo* for drug delivery and many of the studies are still in the simulation stage.

###### Magnetic-based micropumps

**FIGURE 2:**
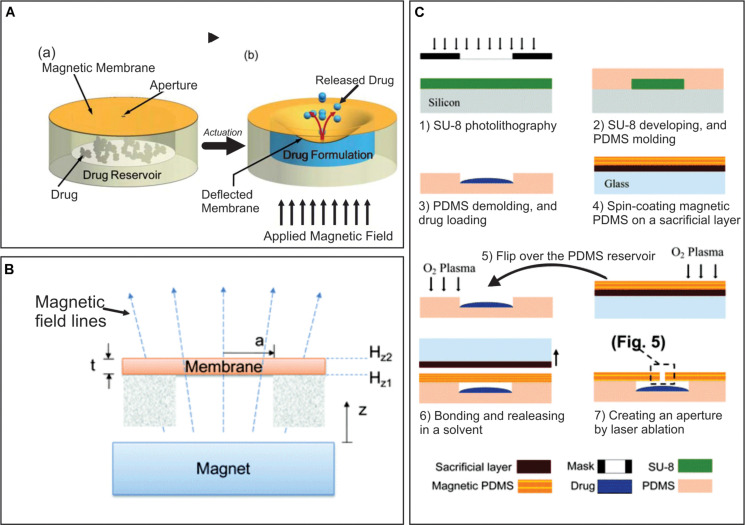
**(A)** Principle of operation, **(a)** before actuation, **(b)** drug release after magnetic field application. **(B)** Schematic illustration of actuation mechanism of a suspended magnetic membrane under an external magnetic field using a permanent magnet. **(C)** Major fabrication steps of a magnetically actuated drug delivery device. Adapted from Pirmoradi et al. (2011a) with permission from The Royal Society of Chemistry.

Micro-electro-mechanical systems drug delivery devices are usually based on embedded batteries for operation and therefore the overall size is conditioned by the electronics and battery size, which limit miniaturization of these devices. In this sense, the development of some drug delivery devices controlled by magnetic forces was reported (Pirmoradi et al., 2011a, b). In one case the device consisted of magnetic PDMS membranes with laser drilled opening sealing micro-reservoirs filled with drug, which were actuated externally by a magnetic field (Figure 2A). The magnetism of PDMS membranes were given by iron oxide nanoparticles coated with a fatty acid surfactant which were dispersed in the polymer. Drug reservoirs were fabricated by molding PDMS using photolithography. Briefly, spin coating of a layer of photoresist was followed by a selectively UV exposition. By pouring of the pre-polymer mix on the patterned silicon substrate and pealing it away from the mold, cavities were generated into PDMS that were ready for drug loading (Figure 2B). The reservoir was bonded to the magnetic PDMS membrane using a sacrificial layer of polyacrylic acid. The magnetic composite membrane was deposited by spin-coating. Surface wettability of PDMS surfaces, which is naturally hydrophobic, was modified by using bovine serum albumin to allow reservoirs filling with drug dissolutions. The actuation was triggered by application of an external magnetic field which concavely deformed the membrane and increased reservoir pressure releasing the drug. Initially, the drug tested was methylene blue owing to its high solubility, high UV-Vis absorptivity which enable and a clear observation of the released drug (Pirmoradi et al., 2011a). The magnetic field for membrane actuation was applied by a rotating permanent magnet. The magnitude of the external magnetic field and the number of actuation events were adjusted to control the amount and the time of released drug. This later feature was determined by an on-demand application of the magnetic field (Figure 2C). In a practically simultaneous study by the same authors, this device was used for controlled release of docetaxel (Pirmoradi et al., 2011b), a chemotherapeutic drug that has antiangiogenic effects at very low concentrations and required a localized administration to treat diabetic retinopathy. Constant and reproducible release rates were achieved over more than a month, with negligible leakage of drug when the device was not magnetically actuated. The biological activity of the drug released from the device was confirmed *in vitro* by analyzing its antiproliferative effect.

**FIGURE 3:**
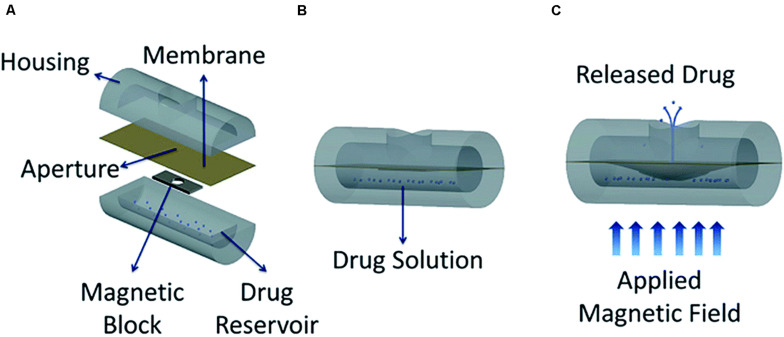
Conceptual diagram of the proposed minimally invasive drug delivery device showing **(A)** device components, **(B)** device under no magnetic field, and **(C)** actuated device under an applied magnetic field. Reproduced from Zachkani et al. (2015) with permission from The Royal Society of Chemistry.

Based on the same principle, a cylindrical MEMS device magnetically actuated was reported as another option. In this study, again two drugs with different solubility in aqueous solutions were studied: the high soluble methylene blue and the low soluble docetaxel, which is also commonly used to treat prostate cancer (Figure 3; Zachkani et al., 2015). The drug delivery device was formed by a drug reservoir fabricated in PDMS, a PDMS membrane, a rectangular magnetic structure and the covering. The modeling was achieved by 3D-printed molds made from a thermoplastic material called acrylonitrile butadiene styrene. The magnetic structure was composed by two layers of iron oxide magnetic nanoparticles between PDMS layers. By the application of magnetic fields, the magnetic structure deflected the membrane and allowed the drug to be release in the covering through an opening, similar to what was initially described by Pirmoradi et al. (2011a). Drugs were then delivered by diffusion through a large opening in the covering. On-demand and reproducible drug release rates were reported to be achieved in this study.

The salient property of these magnetically controlled devices is that they do not need a battery to operate. Instead, they are designed to be actuated by magnetic fields with intensities ranging from 135 to 255 mT. These values are much higher than the magnetic field margins internationally accepted as safe for human health under chronic exposures, which are in the order of μT (IEEE, 2002; World Health Organization [WHO], 2007). Although potential magnetic field damage would depend on the time of exposure, which in this case would be short but repetitive, none of the devices presented in this section were tested *in vivo*, nor the potential harmful effects of the magnetic field application needed for device actuation studied. Another important thing to mention is the complications that would arise with the need for a magnet to activate implanted devices from outside the body, especially in terms of lack of portability and comfort.

###### Phase change-based micropumps

Phase change actuation is based on materials which undergo a phase transition caused by a heating cycle, such as gallium or paraffin. Heat is usually generated through metals micro heaters deposited onto substrates. These micropumps can offer programmable ultra-low flow rates with controllable profiles and low power consumption.

In this context a planar micropump based on gallium phase-change actuation which exerts occlusion of the flow channel through expansion during solidification was described (Johnson and Borkholder, 2016). In this way isolation of the drug reservoir was enabled without consuming energy. The silicon-based MEMS device was 7 × 13 × 1 mm and included fluidic channels, pump chambers, in-plane interconnects and aluminum heaters and contacts. The pump was fabricated by using a combination of MEMS techniques (plasma-enhanced chemical vapor deposition (CVD), plasma etching, wet etching, etc.) and the gallium actuator was produced by additive manufacturing direct write technologies. It was coated with biocompatible Parylene-C, critical for implantable applications. Four actuators were used to propel the fluid peristaltically by transferring momentum to diaphragms made of tetraethyl orthosilicate and Parylene-C. The pump rate was controlled via the frequency of the control circuit and reached a large range (18–104 nL/min) at an efficiency of 11 mJ/nL, which could be theoretically reduced by using a gallium-indium alloy and the addition of a close loop control.

In a similar but more recent micropump design, peristaltic pumping was achieved by expansion and contraction of paraffin wax located in three chambers, which sequentially produced compressing of a microtube (Forouzandeh et al., 2019). The chosen actuation material has a stable phase-change behavior and high-pressure actuation and its melting point can be chosen by selecting the appropriate molecular weight. The mechanical components of the micropump were designed for ultra-low power operation and built using direct-write 3D printing technology around the microtubing, directly on the back of a printed circuit board assembly which provided microprocessor control of actuation and Bluetooth wireless communication. Six resistive heaters and thermistors were used to generate a linear template for three chambers and a groove for the microtubing was placed between them. The overall micropump size was 8 mm × 8 mm × 3 mm. The micropump achieved a resolution of delivery in the range of 10–100 nL/min, in the presence of up to 10× larger than physiological backpressures and by changing the actuation frequency, according to the *in vitro* characterization results. The device also showed biocompatibility both *in vitro* and *in vivo*. It was implanted in mice and results indicated functional sodium salicylate delivery near the round window membrane of the ear. This proof of concept success in mouse which has a cochlea volume of around 620 nL, stimulates translational opportunities. Using appropriate scaling of the microtubing size and actuator volume, the micropump would be scalable for use in larger species. However, device long-term implantation with periodic drug delivery still needs to be studied.

Phase change micropumps offer the advantage of blocking flow in the off state, allowing the drug reservoir to be isolated from the delivery target without consuming energy. They also offer a relative low actuation voltage. Among their main drawbacks slow response time, especially during the cooling process, the low frequency of operation and the limited materials required for actuation should be mentioned.

##### Non-mechanical Micropumps

A non-mechanical micropump exerts forces directly on a liquid and does not involve any kind of structural moving as a part of its operation to flow the fluid. In these systems, a non-mechanical source of energy is converted into kinetic energy (Meng and Hoang, 2012). Non-mechanical micropumps usually have a limited flow rate, slower response compared to mechanical micropumps and often require interaction with a working solution with particular electrical properties, such as conductivity. However, they have shown to be very useful in devices for drug delivery applications owing to their low power consumption. Non-mechanical micropumps can be actuated by electro-hydro-dynamic, magneto-hydrodynamic, electro-osmotic or electrochemical forces, being the last ones the most relevant in drug release applications.

###### Electrochemical-based micropumps

Electrochemical actuation is based on reversible electrochemical reactions. Reduction by electrolysis in an aqueous electrolyte solution enables gas bubble generation and expansion of the reservoir in which that solution is contained. To achieve this, electrochemical-actuator based micropumps include integrated electrodes which are commonly fabricated using photolithography and lift-off process on silicon wafers. These devices usually used titanium/platinum electrodes deposited using an e-beam evaporator (Tng et al., 2013; Liu et al., 2015) or by magnetron sputtering (Uvarov et al., 2016a). In general terms, when a current is supplied to the electrodes, the electrolysis splits the water molecules, generating hydrogen, and oxygen gases (Gensler et al., 2012; Song et al., 2013; Anderson and Davis, 2015). The formation of the gases increases the pressure within the pump chamber, causing the expansion of a flexible membrane and pushing out the contents of the drug reservoir next to the reaction chamber. This mechanism enables drug dispensing, the dose of which would depend on the concentration of drug solution and on for how long the current is supplied to the electrodes. The drugs are prevented from oxidation damage by electrochemical reactions through a separation of the pump chamber and the drug reservoir by a flexible membrane (Tng et al., 2013). Besides, after the current is turned off, the Pt/Ti electrodes catalyze the recombination of gases into water. As a result, a refillable reservoir requires a reduced amount of elements, offering in this way, a sustainable solution to individualized drug delivery.

In this context, an electrochemical micropump composed of metallic electrodes deposited on a glass substrate and a PDMS structure including channels for the electrolyte was reported (Uvarov et al., 2016a). These two parts were manufactured separately and were then connected. Soft lithography technique was used to fabricate the PDMS structure, while deep RIE was utilized for diffusers and channels. Researchers reported two main problems found after preliminary testing of this device. On one hand, after several hundreds of cycles of alternating polarity testing the electrodes underwent fast degradation. On the other hand, the flow rate measured was significantly lower than expected. This was attributed to the softness of PDMS material, as an important part of the produced gas was spent in inflating the chamber instead of pumping. For these reasons, the authors concluded that the chamber of the micropump needed to be fabricated with a stiffer material, as well as the electrodes require choosing an appropriate material more resistant to degradation.

In a subsequent approach, the same research group tested a new version of this device (Uvarov et al., 2016b). Two types of electrodes were used: a platinum electrode and one made of Ti/Al layers. Testing in the alternating polarity mode regime evidenced an increased frequency operation of the pump because of very short gas termination time. Like on the previous assay, the flow rate was lower than expected due to PDMS deformation. While platinum electrodes were fast degraded, almost no damage was found on the aluminum electrodes coated with a titanium layer. Researchers observed that short voltage pulses of alternating polarity generated transient microbubbles as a result of the merge of nanobubbles of H_2_ and O_2_. These microbubbles contain a stoichiometric mixture of gases and ignite spontaneously, liberating enough energy to drive the pump. The flow rate measured in this regime was significantly higher than the one without the formation of these transient microbubbles.

In this context, the same authors continued studying more deeply the dynamics of the gases inside the chamber (Uvarov et al., 2017). As a result of the analysis of this phenomenon, they conclude that the device can be further improved by making some modifications. Firstly, higher voltage amplitude could help to reach a shorter pumping cycle. Secondly, the cylindrical symmetry of electrodes was able to reduce uncorrelated explosions by distributing the nano-sized bubbles more homogenously in the chamber. Thirdly, the operating characteristics could be improved by selecting a more suitable material for the chamber and utilizing aluminum electrodes. They concluded that these exploding microbubbles could be very useful for electrochemical actuators and pumps of varied designs which could include specific bubble force-activated membranes (Huang et al., 2012).

**FIGURE 4:**
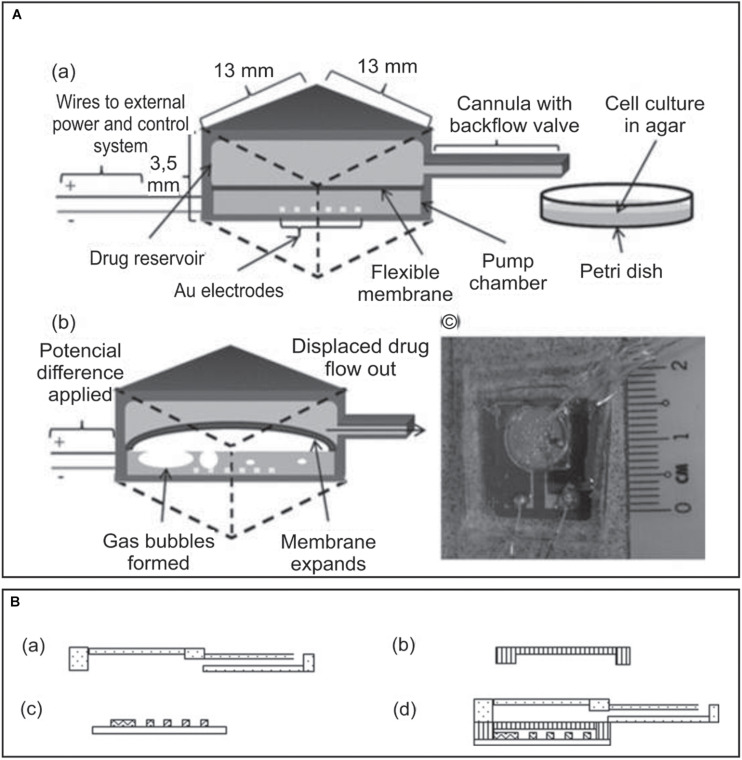
**(A)** Implantable MEMS electrochemical drug delivery device: **(a)** schematic view of the device and delivery, **(b)** electrochemical actuation of the flexible membrane, and **(c)** photo of a prototype model showing the electrode housed within the pump chamber and the drug reservoir lying on top, connected to the cannula. **(B)** Three-part design of the implanted drug delivery system: **(a)** the drug reservoir together with a cannula, **(b)** the flexible membrane, **(c)** the electrodes, and **(d)** fully assembled device. Adapted from Song et al. (2013) with permission from John Wiley and Sons.

Another single reservoir drug delivery MEMS design based on an electrolytic actuator was developed and tested *in vitro* for programmed administration of the chemotherapy drug doxorubicin (Figure 4A; Song et al., 2013; Tng et al., 2013). The device was composed of two chambers: a drug reservoir with a cannula and a pump chamber, the last one containing a set of interdigitated gold electrodes. The fabrication was carried out in three separate parts and components were then assembled (Figure 4B). The electrodes consist of Ti and Au layers deposited by electron-beam evaporation on a silicon substrate. The rest of the device was fabricated individually with PDMS using a micro-machined mold and then connected also using PDMS, which was selected as it is highly biocompatible, cost-effective, flexible, optically transparent and easy to fabricate. A needle syringe was used to fill the drug reservoir with doxorubicin solution, as well as to fill the pump chamber with sterile deionized water, which worked as the electrolyte for the electrochemical actuator. The device was refilled after each test by the same method. Besides, the authors studied the energy losses undergone by the fluid in the transition from the reservoir to the cannula. They simulated the flow and fine-tuned the fabrication in order to minimize the losses. Because of that, the cannula was fabricated at the corner rather than the center of an edge. The device was tested *in vitro* with different programmed administration schedules of doxorubicin using two pancreatic cancer cell lines. As a result, cancer cells growth was successfully inhibited, demonstrating the capability of programmed drug delivery.

Another microdevice, presented by Liu et al. (2015), was fabricated and tested *in vivo* in rodents. The authors made an extensive analysis of previous works to find the most efficient design in terms of size, localized release and re-implantation time. Instead of using multiple smaller reservoirs, they utilized a single large reservoir system in order to resolve the dosing challenge. PDMS molded using SU-8 photoresist was used for the fabrication of a thin-rounded drug reservoir. This design was selected to improve biocompatibility during the insertion of the device, as this shape reduces irritation and extrusion under the skin and tissues. Besides, a long polyolefin cannula was added to the reservoir to avoid the need of implanting the whole device under the skin. A nano-sized sandwiched Pt/Ti multi-layer electrode actuator was utilized in order to enhance the actuation lifetime. Moreover, a thick layer of Ti was settled on the top to protect the electrode from the delamination process. Two thin copper wires were attached to electrodes using silver adhesive conductive paint. The biocompatibility and drug delivery application was assessed by subcutaneous implantation of the cannula on the abdominal cavity of 12 Kunming mice. The evaluated drug consisted of a phenylephrine formulation, which could achieve the expected increase on mice blood pressure and was comparable to traditional administration by the syringe injection method.

**FIGURE 5:**
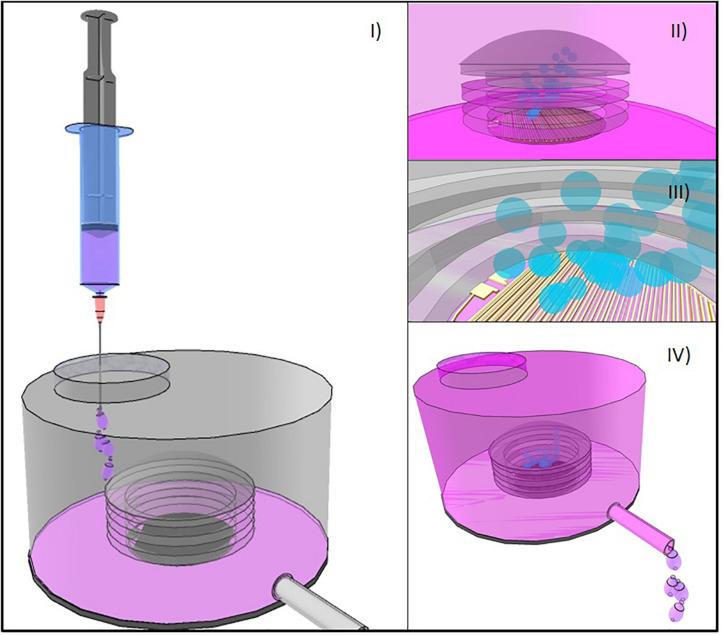
**(I)** Bellows-based electrochemical micropump device with an externally refillable inert reservoir. **(II)** Flexible bellow and the arrangement of electrodes in the inner part of it. **(III)** Electrolysis produces gas release that increases the internal volume of the chamber inside de bellow. **(IV)** As bellows inflate, drug solution is dispensed through a catheter.

An interesting approach in which the electrochemical actuator was based on Parylene bellows containing water and two interdigitated platinum electrodes was described in a series of reports (Gensler et al., 2012; Sheybani et al., 2013a; Cobo et al., 2016). The pressure, increased by application of electrical current to these electrodes, deflected the bellows which were placed inside drug reservoir, turned on a one-way check valve, which was introduced to guarantee appropriate flow regulation, and discharged the drug solution out of the reservoir through an attached catheter to the targeted site (Figure 5). The aim of the bellows was to detach the electrolysis reaction from the drug content to avoid chemical interactions. Dose requirements and desired flow rate for specific applications were used to determine the time the current was applied. In the first of these reports a prototype intended to be used in rodents was described and characterized (Gensler et al., 2012). The device had a refillable drug-containing reservoir made using silicone rubber casting in acrylic molds, a material with broad drug compatibility, ease and inexpensive fabrication and potentiality to avoid irritation and erosion of surrounded tissue due to rounded features. The interdigitated platinum electrodes were deposited by electron-beam evaporation. The optimized Parylene-C bellows had two convolutions, were fabricated on a glass substrate by a molding process involving polyethylene glycol and PDMS and their dimensions was selected for dead volume minimization and maintenance of sufficient drug volume delivery. The bellows actuator was inserted in the reservoir through a slot made on its wall. A biomedical grade elastomer was used to stick the bellows actuator to the base and seal the reservoir. The fully packaged device was cured applying temperature and a needle was used to fill the reservoir. It showed to consume around 3 mW when 1 mA was applied, with a maximum flow rate of *c.a.* 4.7 μL min^–1^. Between delivery pulses, a period of 45 min was needed to allow recombination of gases to water and bellow deflation. This study demonstrated that the fabricated device could be used for drug release in mice with controlled volume and delivery rates. It also showed preliminary results on chronic delivery of anti-cancer siRNA-gold nanoplex-based drug. However, the authors questioned their design in terms of lack of reservoir rigidity, reversed leakage of valves and failure in micropump repeatability.

A similar and improved version of this device was developed by the same research group, utilizing a wireless 2 MHz class D inductive powering system (Sheybani et al., 2013a), and showing to be highly effective for rapid and repeatable bolus delivery. Among improvements, polypropylene was used to fabricate 1 mL-reservoirs and platinum electrodes were coated with Nafion^®^ for repeatable and controlled intermittent pumping. Researchers characterized the performance of actuators under continuous pumping conditions, utilizing several bellows configurations at different currents. They found that the flow rate depended linearly on applied current, so the selection of the current magnitude determined the flow rate. Besides, the actuator operating range was limited by the mechanical properties of the bellows. Furthermore, the recombination of gases into water, which is an imperative factor for repeatable actuation, was also studied for Nafion^®^-coated and bare electrodes. Solubility of oxygen and hydrogen gases in Nafion^®^ was superior to the one in water. As a result, diffusion to the electrode surface was facilitated, hence, recombination was promoted and increased in Nafion^®^-coated electrodes in comparison to uncoated electrodes. This higher electrolysis efficiency, facilitated by the Nafion^®^ coating led to higher flow rates reaching more than 100 μL min^–1^, though increasing the power up to 25 mW. Lastly, real-time pressure measurements were accomplished to investigate how physiological temperature, back pressure, and drug solution viscosity affected delivery performance.

This design was further miniaturized to build an implantable wireless infusion micropump for chronic drug delivery and evaluation of cancer therapies in small research animals like rodents (Cobo et al., 2016). To attempt this, operation was achieved by reducing actuator power consumption to 1 mW by a class E wireless inductive powering transmitter. The device was capable of dispensing up to 185 μL of drug solution and achieved reliable low flow rates in the range of μL/min, which are relevant for cancer therapies. Furthermore, consistent performance was demonstrated for 30 days under a simulated *in vivo* environment considering back pressure, viscosity and temperature, resulting in less than 4% variation, which was appropriate for its function.

**FIGURE 6:**
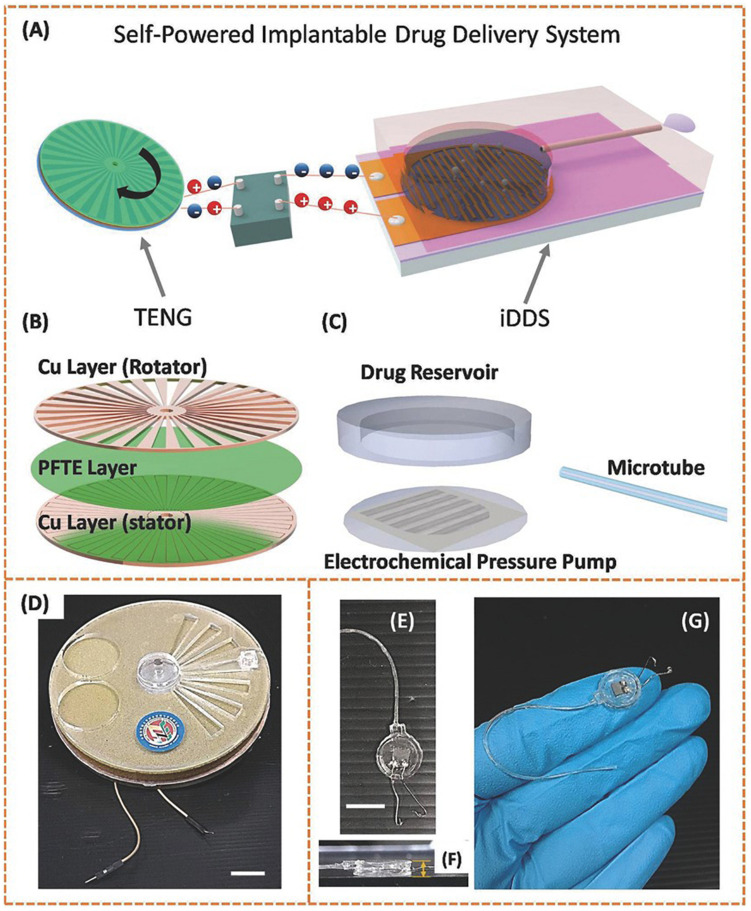
Structural designs and photographs of the TENG and the implantable drug delivery system (iDDS). **(A)** Schematic illustration of the TENG-based iDDS. **(B)** Schematic illustration of the parts in TENG. **(C)** Schematic illustration of the parts in iDDS. **(D)** Photograph of the TENG packaged in glass epoxy. The scale bar indicates 10 mm. **(E)** Photograph of the iDDS. The scale bar indicates 10 mm. **(F)** Side-view photograph of the iDDS. The thickness measured is 2 mm. **(G)** Photograph of the iDDS on a human hand. Reproduced from Song et al. (2017) with permission from John Wiley and Sons.

A recent important contribution within electrochemical actuator-based devices was attempted by a battery-less implantable drug delivery system with the interesting property that powers itself with biokinetic energy (Song et al., 2017). The device consisted of an electrochemical microfluidic pump powered by a triboelectric nanogenerator (TENG). The TENG is capable of using ambient mechanical energy and transforming it into electricity through contact electrification combined to electrostatic induction (Tetteh et al., 2014; Tang et al., 2015). In this work, two layers of copper patterned with radial arrayed strips worked as the rotator and the stator. In between, a layer of poly (tetrafluoroethylene) (PTFE) film represented the electrification material. This TENG was connected to the drug pumping system by wires. The electrochemical pumping system was composed of a PDMS reservoir, a PDMS microtube and a set of gold electrodes on a silicon substrate (Figure 6). Hence, with TENG rotation, the copper rotator slides along the PTFE film, while injecting electrons into the film as a result of the contact electrification. The potential difference generated a current that was then transformed, rectified and applied to the gold electrodes of the pump. The potential on these electrodes split the water, thus hydrogen and oxygen gases pressurize the reservoir, and pumped out the drug contents. The drug release speed correlated with the gas production rate in electrolysis, which was defined by the electrons generation rate in TENG. The researchers measured pumping flow rates from 5.3 to 40 μL min^–1^ at different rotation speeds of TENG. Furthermore, the functionality of the fabricated device for ocular drug delivery was assessed using human hand motion as the power source. The device was tested *ex vivo* in porcine eyes by dispensing a suspension of fluorescent microparticles, which could be imaged for delivery tracing. Therefore, the authors offered here a novel way of self-powering an implantable device, eliminating the need of batteries and making use of biokinetic energy.

Electrochemical micropumps are relatively simple to fabricate and they can be easily integrated with microfluidic systems. They offer a continuous and smooth drug delivery and a large mechanical displacement with low power consumption. Their major limitation is in the possibility of generated bubbles to collapse into water causing an unsteady and unreliable drug release.

###### Acoustic-based micropumps

Recently, a great deal of attention has been focused on acoustic bubbles and acoustically oscillating solid structures, which have been postulated to address some drawback of other pumps, especially to precisely control the flow rate. The applications of bubble-based micropumps have significantly risen in recent years, mainly due to the ability to integrate an acoustic-based pump and to convert the acoustic streaming into a pumping flow. The oscillation of microbubbles generates a flow pattern, which is affected by the gas compressibility and the acoustic force. This phenomena was employed to create efficient micromixers (Lin et al., 2019a), functional particle-polymer composites for accelerated heat dissipation (Lu et al., 2019) or applied to perform single cell analysis in a novel 3D design (Lin et al., 2019b).

Interestingly, a pump that utilizes acoustic streaming induced by localized fluid–substrate interactions can generate stable unidirectional flow (nanoliter/second) with a high degree of precision owing to the developed digital regulation. Moreover, it can handle aqueous and viscous solutions (Wu et al., 2019). Furthermore, Gao et al. (2020), reported for the first time a micropump with the ability to pump fluids in different directions inside a microfluidic device. An interesting feature of this design is that the flow direction was precisely controlled in the device with the frequency and voltage applied to the actuator.

However, there are drawbacks associated with acoustic-based pumps. For example, the bubble’s size would be affected upon time resulting in changes in the resonant frequency of the bubbles. In this sense a great deal of efforts were made to develop theoretical models that would allow the identification of the resonant frequencies and viscous dissipation factors of bubbles (Gritsenko et al., 2018). Finally, the great potential of acoustic-based pumps in terms of high-precision, small volume, possibility to be programmable and low cost allows to envision its great potential for precise drug delivery.

### Multi-Reservoir-Based Devices

Another group of MEMS devices for drug release applications proposes the use of multiple reservoirs in which each reservoir is aim to provide a single dose of the loaded drug (Sutradhar and Sumi, 2016). Different approaches are discussed here based on their actuation mechanisms and summarized in Table 2. In these devices the amount of drug contained in each reservoir is usually completely released in an exact and controlled manner. This is generally achieved by different actuation mechanisms which promote dissolution or rupture of reservoir capping membranes. However, there is a particular case of an implantable multi-reservoir device, in which chemotherapeutic drugs were released passively in order to test tumor *in vivo* sensitivity (Jonas et al., 2015). The device was implanted through a biopsy needle into a tumor, and the drugs were directly delivered into the tumor tissue without systemic exposure. Micromachining of Delrin acetal resin blocks were used for device manufacturing. It consists of 3–30 circular reservoirs that release the drugs passively into distinct regions of tumor tissue for 24 h. Appropriate spatial separation of the reservoirs avoided adjacent drugs overlapping. Researchers managed to control the release profile of drugs through several techniques: modification of the reservoir opening size, use of a polymer matrix in drug formulation to control the diffusion properties in the tumor, and hydrophilic hydrogels with expansive capability to eject the drug from reservoirs into the tumor tissue. Furthermore, a combination of drugs could be tested by loading them into the same reservoir. Thus, researchers obtained a device capable of testing up to 16 drug combinations, which can be used to define which would be optimal drug therapy before starting a systemic treatment.

**TABLE 2:** Multi-reservoir-based MEMS devices for drug delivery applications.

Actuation mechanism	Device dimensions	Drugs tested	Main materials	References
NIR irradiation	27 mm × 11.5 mm × 9.5 mm (length × width × height)	Human growth hormone	MEO2MA-co-OEGMA (POSS) Graphene oxide nanoparticles Polyurethane Medical epoxi Parylene C	Lee et al., 2019
Passive release	820 μm (diameter) × 3 mm (length)	Chemotherapeutic drugs: doxorubicin, sunitinib, lapatinib, antibody cetuximab, dasatinib, gemcitabine, paclitaxel, cisplatin	Medical-grade Delrin acetal resin blocks	Jonas et al., 2015
Electrothermal	(13 × 5.4 × 0.5) mm	Human parathyroid Hormone fragment (1–34) [hPTH(1–34)]	Titanium housing Silicon wafer Ti-Pt	Farra et al., 2012
Electrothermal	15 mm × 15 mm × 1 mm	Leuprolide	Titanium housing Silicon wafer Pt/Ti/Pt membranes	Prescott et al., 2006
Electrochemical	N/S	1,3-bis(2-chloroethyl)-1-nitrosourea (BCNU)	Silicon wafer Au Pyrex	Li et al., 2005
Electrothermal	N/S	Mannitol	Silicon wafer Au or Pt/Ti/Pt membranes	Maloney et al., 2005
Electrochemical	(17 × 17) mm × 310 μm	Sodium fluorescein and ^45^Ca^2+^ (as CaCl_2_)	Silicon wafer Au SiO_2_	Santini et al., 1999

*N/S, not specified; Au, gold; Pt, platinum; Ti, titanium; Cu, copper; PDMS, polydimethylsiloxane: PO, polyolefin; PTFE, poly(tetrafluoroethylene); PZT/PZT-5H, piezoelectric materials; MDX4-4210, biomedical grade silicon elastomer; SiO_2_, silicon dioxide.*

#### Electrochemical Dissolution of Reservoir Capping Membranes

Electrochemical dissolution is itself an electrolytic process. In this technique direct electric current is applied externally to drive a non-spontaneous chemical reaction. In MEMS devices this reaction offers an effective controlled opening of reservoirs that can be easily used on drug release applications. Thin anode membranes cover micro-reservoirs which are filled with drugs to release. The release mechanism is based on membrane corrosion processes which allow the delivery of specific doses of the drug under study which is loaded in those reservoirs (Zhang et al., 2017).

A solid-state-multiple-reservoir silicon microchip with no moving parts able to provide controlled release of single or multiple drugs on demand through the electrochemical dissolution of capping membranes was first described by Santini et al. (1999). The main objective of this initial study was to determine if the pulsatile release of model chemical compounds could be obtained from the microchip device containing 34 reservoirs. However, the authors stated that device size (17 mm) had enough surface area to hold until 1,000 reservoirs and that it could be reduced depending on the application. Devices were fabricated using silicon wafers and microfabrication processing techniques including photolithography, electron beam evaporation, CVD, and RIE. By these, reservoirs that went through the width of the wafer were generated. The reservoirs had a square pyramidal shape, with a volume of 25 nL, and were sealed on their smaller end by a thick gold membrane anode. Ease of deposition and patterning, low reactivity and corrosion resistance were the factors that guided the election of gold as a model material. However, the formation of soluble gold chloride complexes favored by the presence of a small amount of chloride ions has been observed. For reservoirs filling, inkjet printing with a computer-controlled alignment or microsyringe pumps were used to deposit 0.2 nL of an aqueous solution of a compound and a liquid polymer into each one. The water was evaporated after injection and only liquid polymer and model compound remained in the reservoir. Release from the reservoir was initiated by applying an electric potential between a cathode and the gold anode membranes over the reservoirs and was based on the electrochemical dissolution of this thin anode. Parts of electrodes were protected from unwanted corrosion produced by the environment by an isolating silicon dioxide coating produced by plasma-enhanced CVD and characterized by an adequate density and adhesion to the gold deposit. This study demonstrated that the opening of each reservoir can be individually triggered and that varying amounts of chemicals can be released in a pulsatile or continuous and sequential or simultaneous manner from a single device. The biocompatibility and biofouling of the proposed microfabrication materials were demonstrated *in vivo* (Voskerician et al., 2003). However, the potential harmful effects of soluble gold chloride complexes formed upon dissolution of reservoir capping membranes was not evaluated.

In another work, a multiple-reservoir device was used to release carmustine [1,3-bis(2-chloroethyl)-1-nitrosourea (BCNU)], a drug used for brain tumors treatment, by an electrochemical-dissolution-based mechanism (Li et al., 2004, 2005). The drug was co-formulated with polyethylene glycol to enhance release kinetics. The device was based on 20 micro-reservoirs etched into a silicon substrate and bonded to a Pyrex structure containing two macro-reservoirs with a volume capacity of approximately 9 μl. Each macro-reservoir was physically connected to 10 micro-reservoirs in order to increase the device capacity. The materials and process for microfabrication, as well as the actuation mechanism, were the same as described by Santini et al. (1999). Two drug release events could take place each time 10 micro-reservoirs were opened by dissolution of their gold capping membranes to enable the release of each macro-reservoir contents. The study proposed a performance comparison between the local delivery of BCNU from devices, which were implanted next to experimental gliosarcomas in rats, and subcutaneous injections of the same drug by evaluating the tumor growth *in vivo*. The results indicated that the device activation process was not detrimental for released BCNU activity and therapeutic benefit. However, the fact that only two release events can be accomplished constituted an important limitation of this particular design.

**FIGURE 7:**
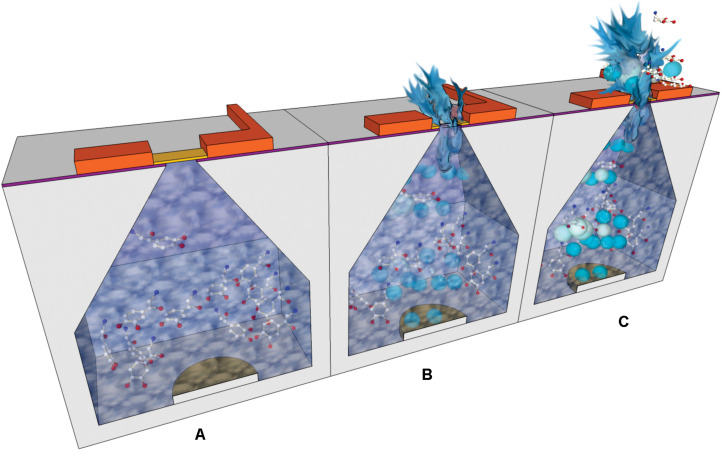
Cross-section schemes showing the operational steps of an electrochemically actuated drug delivery device: **(A)** Drug dissolution is loaded in the reservoirs; the electric potential is applied between top and bottom electrodes. **(B)** The gold capping membranes are dissolved and the electrolysis of water caused gas release. **(C)** The generated bubbles drive the outflow of reservoir contents.

An implantable silicon micro-reservoir device that enables drug delivery in a controlled manner using a robust electrochemical mechanism was reported by Chung et al. (2009). In this case the electric potential was applied between the reservoirs capping membranes and another electrode at the inside bottom of the reservoir (Figure 7). This caused gradual rupture and opening of the capping membranes by gold dissolution. The electrolytic reaction produced gas release inside the reservoirs which forced the contents to go out of them. With this design, less time was required to eject the content out of the reservoirs, compared to other devices that operate on the same principle. Also, this device enabled delivery of higher volumes thanks to the inclusion of Pyrex-PDMS patterned macro-reservoir extension placed behind the silicon chip. Parylene coating of PDMS was applied to avoid loss of fluid through the PDMS substrate. In terms of power requirement, around 5 mW were required for an ejection rate of *c.a.* 4 μL/min. However, *in vivo* testing of this design remained pending and the authors suggested applying a surface modification with polyethylene glycol to minimize physiological response after implantation.

#### Electrothermal-Based Actuation

The electrothermal activation process involves metal membranes which are subjected to an applied current. The generation of local resistive heating causes failure of the membrane and, therefore, the release of the drug reservoir content. Preferential heating on the membranes is due to several reasons. The environment around the suspended membrane possesses lower thermal conductivity than the substrate. Furthermore, the material constitution of the membrane may be more resistive than the trace material, thus increasing heat generation. Besides, membrane area is smaller than those of the traces, which increases the current density and heating. In this way, membrane failure occurs as fast as 5 μs after current application.

The theory and characterization of this actuator was demonstrated by Maloney et al. (2005), who described the first electrothermally activated microchip. The device was adapted from the electrochemically actuated microchip previously described by Santini et al. (1999), which was the first controlled release microchip reported. The electrothermally activated device consists of multiple reservoirs sealed and actuated individually. In this study, two types of membranes were assessed: gold or three layers of titanium and platinum (Pt/Ti/Pt). Gold and titanium materials were selected to exemplify relatively low and high resistivity materials. Platinum is an inert, noble material, and had the purpose to protect Ti *in vivo* and during fabrication, when several RIE etching steps were used. As it was expected, researchers demonstrated that less current was needed to rupture the Pt/Ti/Pt compared to the gold membranes. This was justified to be the result of the much higher resistivity of Pt/Ti/Pt. *In vitro* release of ^14^C-labeled mannitol demonstrated that this technology is reproducible and robust. In addition, researchers suggested that this device may be used for biosensors that are susceptible to failure caused by biofouling.

**FIGURE 8:**
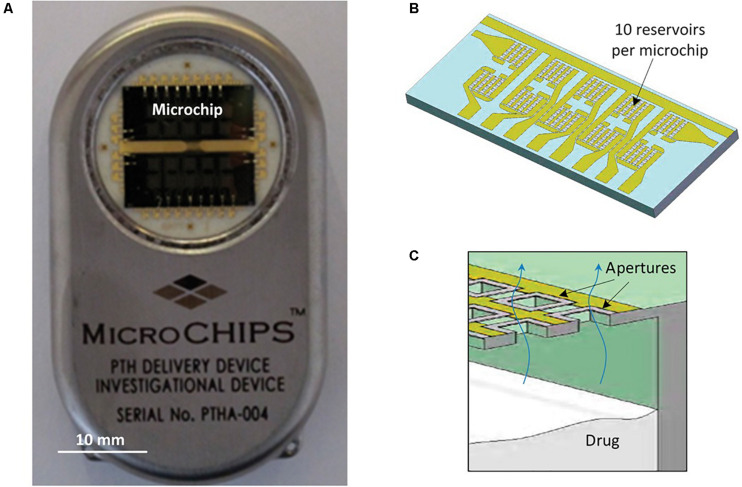
The microchip-based drug delivery device. **(A,B)** Microchip based hPTH(1–34) drug delivery device (54 mm × 31 mm × 11 mm, l × w × h) **(A)** containing two microchips with 10 reservoirs each (13.0 mm × 5.4 mm × 0.5 mm, l × w × h) **(B)**. **(C)** Schematic cross section of microchip assembly showing drug releasing from one reservoir. Reproduced from Farra et al. (2012) with permission from The American Association for the Advancement of Science.

These electrothermally activated microchips were connected to a wireless communication system in order to obtain an implantable device for controlled pulsatile drug delivery (Prescott et al., 2006). In this case microchips contained 100 individual reservoirs of 300 nL each. The release study involved the peptide leuprolide acetate, which is an analog of a luteinizing hormone-releasing hormone, used for the treatment of endometriosis and prostate cancer. The lyophilized leuprolide was formulated in a matrix of solid polyethylene glycol. Indium tin eutectic solder were used to seal reservoirs by thermocompression bonding. The resulting device was implanted subcutaneously in beagle dogs and peptide release was maintained for 6 months. Leuprolide bioavailability and the release kinetics parameters were constant for the whole period. As a conclusion, loading and *in vivo* releasing of a solid-phase drug formulation can be fulfilled and controlled by telemetry. The small volume that can be contained in the reservoirs could be a limitation for this microchip. As a result, researchers focused on the study of drugs sufficiently potent to make them compatible with the volumetric constraints of the device. Among these drugs, human parathyroid hormone hPTH is of great interest as a therapeutic agent for osteoporosis. Proos et al. (2008) successfully prepared clinically efficacious doses of the 34-amino acid N-terminal fragment of the hPTH, hPTH(1–34), in a lyophilizable solution phase form that could be contained in the reservoirs. They demonstrated long-term stability of this dosage form and consistent, pulsatile release kinetics was assessed *in vitro*. This device was further evaluated *in vivo* in a clinical trial (Farra et al., 2012). Devices containing two microchips and a total of 20 doses of lyophilized hPTH(1–34) were implanted in osteoporotic postmenopausal women for several months and wirelessly programmed to release daily doses (Sheybani et al., 2013b). Each microchip included 10 reservoirs (of 600 nL) containing 40 μg doses of hPTH(1–34). A membrane composed of titanium and platinum was utilized for electrothermal activation, as previously described by Maloney et al. (2005). The 20 membranes were connected to metal contacts, which were used as the path for current pulses to melt the individual membranes, thereby exposing the contents of the reservoirs to the surrounding tissue fluid and promoting drug release. Micro-reservoirs were aseptically filled with the soluble drug formulation which was further lyophilized. To provide hermetic seal to microchip assemblies a compression welding process at room temperature was utilized, as this was critical for preventing drug degradation. Hermeticity tests were performed to select the devices for implantation. The dosage release was programmed using wireless control and dose scheduling was wirelessly transmitted by the programmer. The wireless communication connection allowed knowing implant status information, such as embedded battery voltage and delivery confirmation (Figure 8). This type of devices could be benefited by the near-field capacity coupling for wireless power transmission by which hundreds of milliwatts of power can be transmitted safely to the implanted device (Jegadeesan et al., 2017). This was the first in-human study to prove that electrothermally activated microchips are capable of programmable dosage release using wireless control. One of the aims of the study was to evaluate the clinical relevance of the fibrous capsule formed around the implant, specially whether it modifies the pharmacokinetic profiles of hPTH(1–34), as it was presumed to affect diffusion of the drug across the membrane. The study demonstrated that the device containing hPTH(1–34) drug combination was biocompatible, was well tolerated by the patients and was not causal of adverse immune reaction. The pharmacokinetic profiles from the implant were similar to the profile of multiple subcutaneous injections, in spite of the capsule surrounding the implant. Besides, bone formation was stimulated by the implanted device, evidenced by an increase in the bone formation marker type I collagen propeptide. Although this device contained micro-reservoirs only for up to 20 doses, a larger number of reservoirs would be needed to deliver daily doses of other drugs for 1 or more years. The cost of this drug delivery device implanted to provide a medication over the course of 1 year was expected by the authors to be the same as other implantable electronic devices, such as pacemakers and cardioverter defibrillators.

#### Infrared Radiation-Based Actuation

The use of actuation mechanisms based on non-invasive infrared radiation began to be investigated very recently. In this sense, stimulus responsive membranes were used for capping drug reservoirs in an implantable device (Lee et al., 2019). These membranes were made of thermosensitive polymer hybrid [POSS (MEO2MAco- OEGMA)] and photothermal nanoparticles of reduced graphene oxide. Drug reservoir body, top and bottom covers were fabricated with a PolyJet 3D printer using polyurethane copolymer and medical epoxy glue was used to assemble the constituent parts of the device.

**FIGURE 9:**
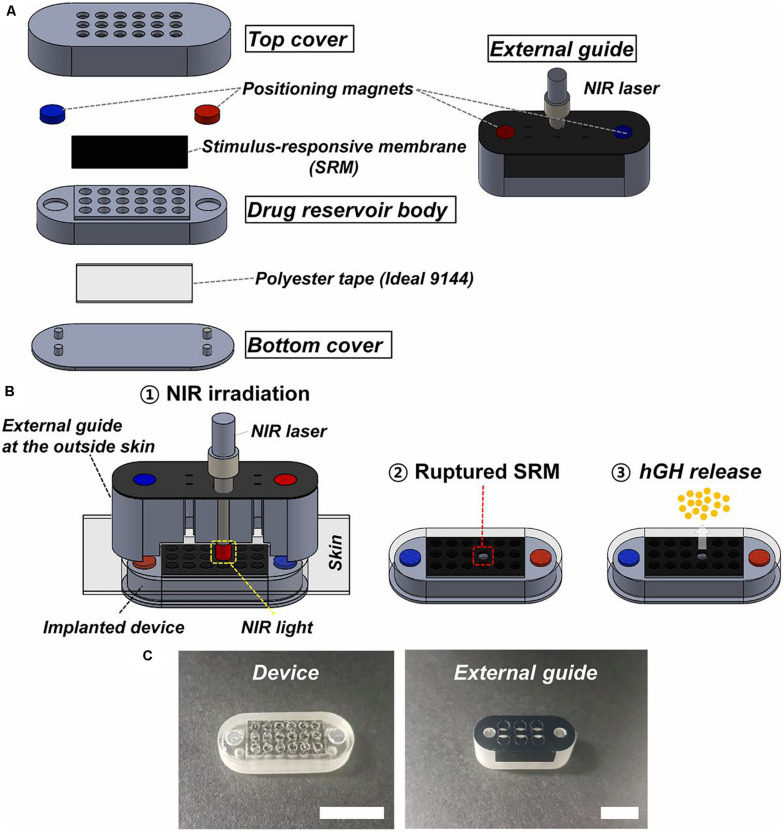
Description of the device and external guide. **(A)** Three-dimensional schematic of the device and external guide. **(B)** Working principle of the device: 1 – the near-infrared light is irradiated through the external guide aligned with the implanted device; 2 – the stimulus responsive membrane capped on the selected drug reservoir is ruptured and opened; and 3 – the hGH is released. **(C)** Optical images of the device and external guide (scale bar: 1 cm). Reproduced from Lee et al. (2019) with permission from the National Academy of Sciences of the United States of America.

The device was 24 mm in length, 9 mm in width, and 5 mm in height, occupying a volume of 1 mL approximately. The prototype arrangement contains 18 drug reservoirs (Figure 9). Each drug reservoir was 1.5 mm in diameter and 3.5 mm in height, having a volume of around 6.2 μl. The actuation for drug release used non-invasive near-infrared irradiation (808 nm) from the outside skin, which offered high penetration and no tissue damage. This irradiation produces graphene oxide excitation that generates heat to break the responsive membranes. This device was tested with human growth hormone (hGH), which needs a pulsatile and on-demand delivery regime. In the implanted device a single and specific drug reservoir could be open to release a quantity of hGH. Parylene C was used to coat the whole surface of the device to promote its biocompatibility after implantation. Each reservoir contained 20 μg of recombinant hGH and polyethylene glycol which increased its stability. An external guide for alignment of the infrared irradiation from the outside body was also fabricated. The device and external guide were magnetically attached with directionality. The membrane was aimed to be ruptured just with a 5 s irradiation. To test device performance *in vitro*, the responsive membranes were irradiated with near-infrared light while immersed in buffer at 37°C for 28 days. To test the *in vivo* efficacy of hGH delivery, the device was implanted subcutaneously in hypophysectomized rats and irradiated on the outside skin once per day for 14 days after implantation. Although a slight difference was observed in the time when the maximum hGH concentration in plasma was reached compared to conventional injection treatment, both hGH and insulin-like growth factor (physiologically secreted in response to hGH stimulation) plasma concentrations were not different between the subcutaneous injection and device groups at any tested time.

Nowadays, most devices contain the active driving units and their power source integrated with the drug reservoirs, making its implantation difficult due to being heavy and bulky. The proposed device herein sought to improve these aspects by offering a promising and minimally invasive strategy for on-demand and pulsatile drug delivery, based on light activation. Near infrared light wavelength range between 650 and 950 nm (this device used 808 nm), is considered as one of the optical windows in biological tissues, also known as therapeutic windows (Weissleder, 2001; Lane et al., 2018). However, the activation process to open the reservoirs could appear uncomfortable for human application, since it supposes the need for a light source localized outside the body.

## Transdermal Drug Delivery

### Needle-Based Transdermal Drug Delivery Devices

When poor drug absorption or enzymatic degradation in the gastrointestinal tract or liver turn oral administration of drugs not feasible, alternative drug delivery systems need to be considered. An approach that is appealing to patients, remaining painless as compared to intravenous and intramuscular injections and offering an easy self-administration and the possibility of controlled release over time, is the non-invasive drug delivery across the skin using a patch. In this sense, transdermal drug delivery (TDD) systems has been being researched since decades seeking for high bioavailability, controllable plasma levels, and less overall dose (Rosen et al., 2017; Szunerits and Boukherroub, 2018). However, the TDD has been greatly limited since most drugs, especially macromolecules, cannot enter the skin in therapeutically useful doses. Different approaches, such as the use of chemical enhancers (e.g., lipids, glycols) or physical methods (e.g., iontophoresis, electroporation, ultrasound) have been explored to increase skin permeability. Beyond their differences, these methods share the aim of creating pathways of sufficient size for drug molecules to go through the *stratum corneum*. These are large enough to permit transport of small drugs and, in some cases, macromolecules, and small enough to prevent damage of clinical significance (Prausnitz, 2004). These approaches, however, have shown critical weaknesses. Physical methods require additional equipment and conventional TDD systems using chemical enhancers have failed in providing a precisely self-controlled pharmacological behavior and high delivery efficiency due to absorption and permeation limitations.

**FIGURE 10:**
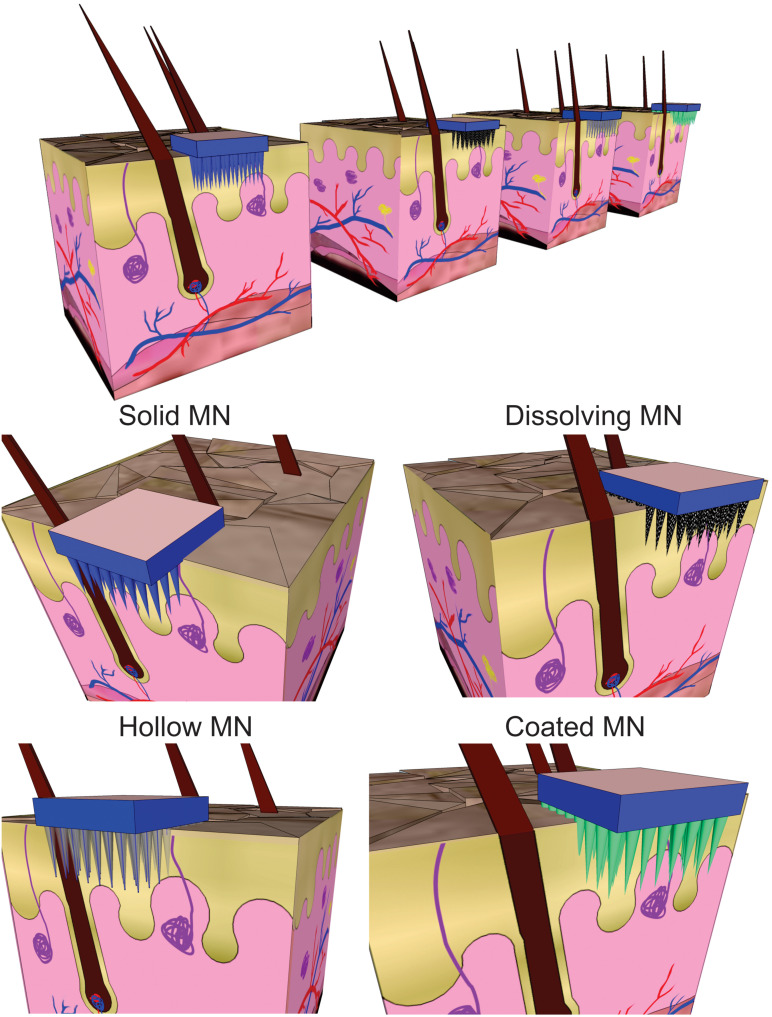
Schematic view of different strategies for transdermal drug delivery using microneedles (MN).

An approach involving the generation of larger transport pathways through skin by using arrays of microscopic needles (with a length lower than 1 mm) as TDD MEMS was suggested to address these issues by improving drug delivery efficiency and control (Indermun et al., 2014). The motivation for microneedles (MNs) was generally related to the fact that they can offer minimally invasive facilitated transport of macromolecules into the skin by enhancing its permeability, while remaining safe, painless and liable to be self-administered. They can also offer site-specific drug release with a reduced dosing frequency and maintenance of constant drug concentrations. MEMS based TDD systems are mainly based on the use these structures and different approaches are used when applying these structures for drug delivery (Chen et al., 2016). In the case of solid MNs the skin is pretreated and then the drug diffuses through the pores in the skin from a topical drug delivery system. When drug-coated MNs are used, after insertion, the coating containing the drug dissolves in the skin. Another category is formed by arrays of aqueous-soluble MNs which perforate the skin and release the drug after MN dissolution. Meanwhile, hollow MNs are like conventional needles but shorter and are used to deliver liquid formulations (Figure 10).

An interesting design of a TDD system using modular reservoirs for drug storage was fabricated and characterized (Cantwell et al., 2014). The device is composed of two parts, a modular reservoir constituted by single-use disposable cartridge and a main structure with integrated MEMS-based drug delivery system. The idea is that prior to attaching to the skin, the filled modular reservoir and main part would be manually assembled. The main structure had several components: an upper hollow MN which was connected to the modular reservoir through a septum on assembly, a lower hollow MN array which enabled minimally invasive TDD, micropumps, valves, control circuitry, and power source, which enable controllable extraction of drug loaded in the modular reservoir. The modular reservoir was formed by a silicon interface plate, a PDMS membrane and an acrylic cap. The materials were carefully selected. The factors that guided the use of silicon included rigidity and versatility for batch microfabrication. PDMS was selected owing to its elastomeric nature, its optical transparency, micromolding capacity, and compatibility with drug formulations. From its part, acrylic offered optical transparency, rigidity, machinability, and low vapor permeability. The interface plate was based on a silicon core surrounded by thin PDMS layers and was a rigid enclosure for the loaded drug and included PDMS-based septum for filling and draining of the modular reservoir by a hollow MN. The acrylic cap contained a vent hole which was sealed with polyimide tape after the reservoir was filled. This modular reservoir was designed to load 400 μl of insulin for a 12-h delivery but the authors considered this design could be adjusted to other fill volumes. Salient features of this modular reservoir concept included reproducible delivery and very low fluid loss during the storage.

Interestingly, some fundamental studies analyzed changes in mechanical forces and moments of silicon MNs when inserted in soft tissues, for their effective and safe use (Rajeswari and Malliga, 2014, 2015). This mechanical interaction was shown to be affected by forces and moments in diverse directions, as well as mechanical and geometric properties of MNs itself. An analytical model allowed understanding the effect of microneedle geometry on the necessary force to insert MNs into the human skin. The performed analyses suggested that the optimal length for silicon MNs is around 300 μm and that smaller lengths would reduce the pain during insertion. This study particularly described interfacial contact forces following the puncture event and evaluated the stresses exerted on the MN and highlighted that understanding of proper modeling of fracture resistance and failure mechanics is vital to improve the safety margin. In this regard, it is to note that microneedle optimization scaling is strongly dependent on multiple parameters such as fabrication materials, skin thickness and action area (Al-Qallaf and Das, 2009; Chen S. et al., 2020).

More recently, an interesting approach described the fabrication of long (158 μm) and tapered solid silicon MNs, achieved by optimization of etching process using tetramethylammonium hydroxide (TMAH) (Narayanan and Raghavan, 2017). Variation of reactive concentration, etching time and rates, temperature, and optical mask design were considered. Among them, the temperature and the position of the sample in the etching solution showed to be of critical importance to obtain high aspect ratio MNs. Hardness tests revealed that the fabricated MNs satisfied the requirements of mechanical stability to penetrate into the skin.

**FIGURE 11:**
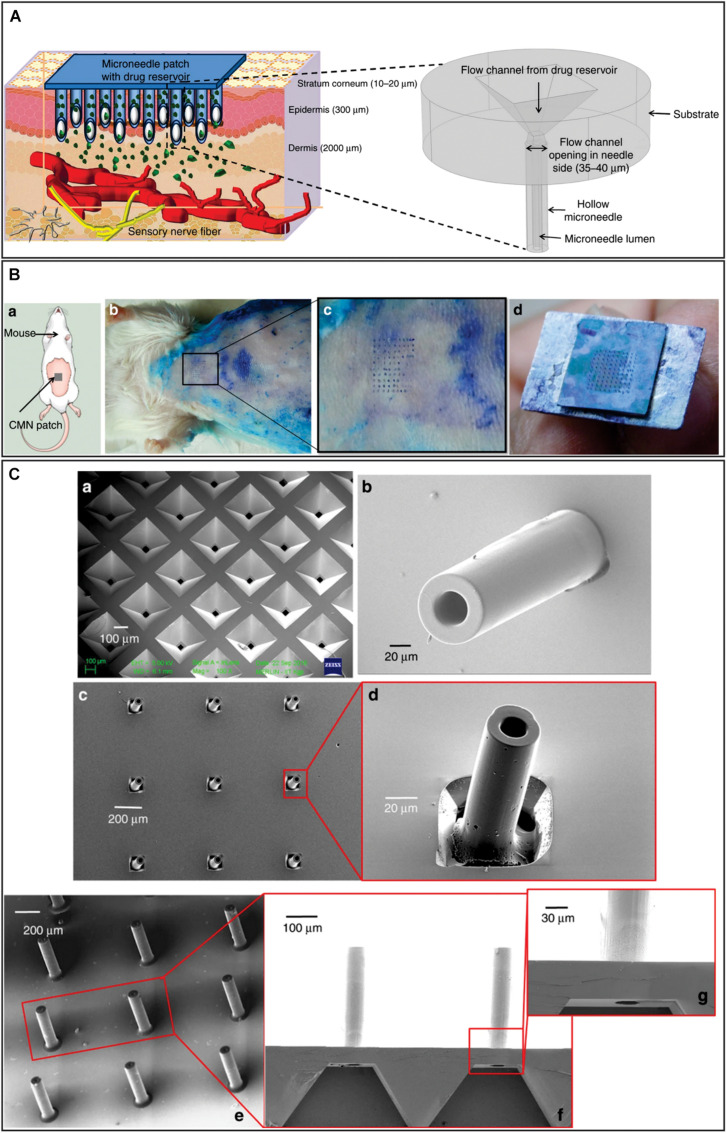
**(A)** Hollow microneedles puncture the skin to reach above the pain-sensing nerves in the transdermal region of the skin and painlessly release the drug through suitable actuation methods. The figure in the outset shows a magnified view of the microneedle structure proposed in this work. **(B) a:** schematic of the microneedle insertion test on mice; **b:** biological insertion test performed on 6- to 8-week-old Swiss Albino mice; **c:** magnified view of the skin area pierced by the glassy carbon microneedles; **d:** intact array of 10 × 10 after multiple insertions. **(C) a:** Etched microfluidic conduit in silicon through which the drug flows from the drug reservoir to the glassy carbon microneedles; **b:** SU-8 microneedles fabricated on a microfluidic conduit backside of the image shown in **a**; **c:** glassy carbon microneedles array formed after pyrolysis; **d:** magnified view of a glassy carbon microneedles; **e:** optimized glassy carbon microneedles aligned on etched microfluidic ports on a silicon wafer. Adapted from Mishra et al. (2018) with permission from Springer Nature (http://creativecommons.org/licenses/by/4.0/).

Materials other than silicon were also considered for MN fabrication. One of these studies described the fabrication and analysis of hollow and tubular hafnium oxide MN. Their fabrication was based on deep reactive ion etching and atomic layer deposition, and required only one mask (Zhang et al., 2018a). This study mainly analyzed MNs flexibility and concluded that this feature could be tuned depending on the required application. Another relevant studies, reported the design, fabrication and characterization of a hollow glassy carbon MN array (Pramanick et al., 2016) with flow channel integration (Mishra et al., 2018). The strategy was based on the direct laser writing patterning of photoresist as precursor and conversion of these structures into glassy carbon by pyrolysis retaining their tubular MN shape. These MNs were 500 μm for long, had an outer diameter of 100 μm, and an inner diameter of 40–90 μm (Figure 11). The maximum forces carbon MNs can endure showed to be approximately two orders of magnitude higher than the resistive forces presented by the skin. When a MN array was inserted into mouse skin multiple times, it was successfully removed without the breakage of any MN. Microfluidic conduits were fabricated in a silicon substrate by conventional wet chemical etching and fabricated MNs were aligned with the reservoir outlet. A cost-effective system to deliver drugs to patients in a precise and painless manner using this design would need the combination of the MN array with a micropump and drug reservoir.

Although many materials such as silicon, metals and glassy carbon have been used for MN fabrication and were tested *in vivo* in the past (Mikszta et al., 2002; Martanto et al., 2004), these failed on their biodegradability and exhibit some safety issues. Instead, polymers [e.g., hyaluronic acid, poly(lactic-co-glycolic acid), gelatin methacryloyl], offer solutions to these issues, also being their use in the fabrication of MNs simpler and more cost-effective than that of the aforementioned materials. The geometry, as well as chemical and physical properties of polymeric MNs can be fine-tuned for optimal monitoring (Zhu et al., 2020), regeneration therapies (Lee et al., 2020), or TDD applications (Singh et al., 2019).

Several techniques have been proposed for fabrication of polymeric microneedles, being micromolding, drawing lithography, electro-drawing and droplet-borne air blowing among the most widely used. Micromolding involves replication of a master template which can ultimately yield many MN arrays following several steps. Use of heat or UV light during the procedure can critically affect the loaded drugs. However, it is a simple, reproducible and scalable process and some improvements have been recently reported (Chen Y. et al., 2020). Drawing lithography is a direct patterning process based on viscosity and elastic deformation of polymers in glass transitions, thereby avoiding the need of mask and light irradiation but requiring polymer curing and with limitations when biodegradable materials are intended to be used (Lee and Jung, 2012). Electro-drawing is another mold- and UV-free technique, which adds the contact-free feature and uses an electrohydrodynamic driving force in mild-temperature conditions. It has been useful for MN fabrication using biodegradable materials (Vecchione et al., 2014; Ruggiero et al., 2018). Droplet-borne air blowing is another fast and mild-temperature fabrication process in which MNs are formed by air blowing and drying of microdroplets contacting two surfaces while these are being separated (Kim et al., 2013).

Most recent contributions of polymeric MNs in TDD applications include the sustained release of contraceptive drugs (e.g., progestagens) by either a drug-loaded polylactic acid and poly(lactic-co-glycolic) acid microneedle patch coated by a drug-free polymer film (Li et al., 2019) or by a multiple-layer MN patch for slow dissolution (He M. et al., 2018; Donnelly and Larraòeta, 2019). Prolonged release of chemotherapeutics (e.g., doxorubicin) was also achieved by drug loading into gelatin methacryloyl MNs using one molding step (Luo et al., 2019). Burst drug release was also possible by using highly dissolvable MN patches. Examples of this are the rapid release of acyclovir for the treatment of herpes labialis (Pamornpathomkul et al., 2018), of dihydroergotamine mesylate for the management of acute migraine (Tas et al., 2017) and of vitamin K during bleeding (Hutton et al., 2018). When targeted release was sought stimuli-responsive MN systems could offer alternatives too. MN patches fabricated using a hydrogen peroxide-labile copolymer encapsulating insulin and glucose oxidase, were able to release insulin under hyperglycemic conditions (Zhang et al., 2018b). In contrast, enzyme-free glucose-responsive MN array for insulin delivery was achieved by a using boronate hydrogel and biocompatible silk fibroin for fabrication (Chen et al., 2019). Another example of targeted delivery was the delivery of STAT3 siRNA for the treatment of melanoma by dissolving polyethylenimine MNs (Pan et al., 2018). Polymer MNs have also been used recently for vaccine delivery. A MN patch layer-by-layer coated with a synthetic pH-induced charge-invertible polymer was successfully used for a rapid release of an antigen and induction of an elevated immune response (He Y. et al., 2018). Enhanced cancer immunization was achieved by dissolving MNs based on an amphiphilic triblock copolymer, which generated nanomicelles containing a model antigen (OVA) and a vaccine adjuvant (R848) upon insertion in the skin and dissolution (Kim et al., 2018). Photothermal activation was also applied for induced release of the antidiabetic drug metformin which was incorporated with bismuth nanodots in poly(vinylpyrrolidone) dissolving MNs (Liu et al., 2018).

Biodegradable polymeric MNs can be effectively activated for drug release by exploiting the polymer and encapsulated drug properties. Their efficiency, cost-effectiveness and the ease of self-administration by patients must be highlighted. However, their precise and uniform large-scale manufacturing remains a challenge and requires further technological innovation.

### Needle-Free Injectors for Drug Delivery

**FIGURE 12:**
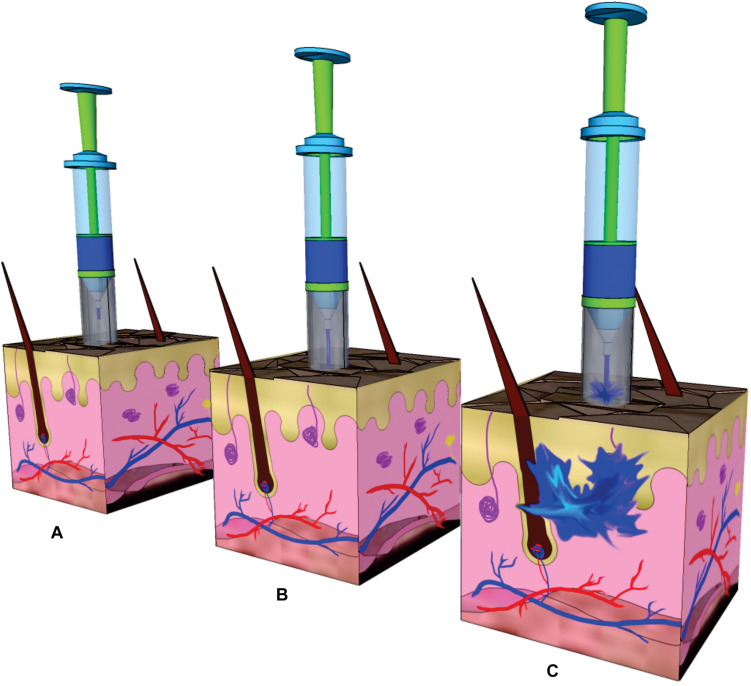
Needle-free injection process: **(A)** Location of the device in the selected area. No need for needle insertion into the skin. **(B)** Shooting of the jet out of the nozzle at high speed (>100 m s^–1^) owing to the impact of a piston on a liquid reservoir. The impact of the jet on the skin surface forms a hole in the skin by erosion or fracture. **(C)** The continuous impact of the jet increases the depth of the hole in the skin. The liquid that has accumulated in the hole slows the incoming jet. The liquid disperses in the skin due to the stagnation of the jet at the end of the hole.

Needle injection has been one of the main drug delivery mechanisms for intravenous, intramuscular or subcutaneous administration of drugs. However, it exposes some disadvantages like no compliance to the treatment, a degree of phobia, damage and pain caused by injection, among others. All these stimulated research to generate new and more efficient strategies (Berrospe-Rodriguez et al., 2016). In this context, in addition to microneedle-based technology previously exposed, some needle-free systems also aimed at solving these problems were developed. Needle-free jet injectors are currently one of the effective alternatives to conventional needle injections, with multiple commercial devices cited in clinical trials (Bremseth and Pass, 2001; Yousafzai et al., 2017; Bavdekar et al., 2019). TDD based on this mechanism entails ejection of a liquid drug through a fine nozzle at elevated pressure, thus generating a narrow super-fast fluid jet easily penetrable into the skin and tissue (Figure 12).

In the literature, researchers have explored different actuation mechanisms to generate a steady and high speed jet. In this sense, the design and simulation of a simple and compact piezoelectric actuated needle free injector which offers control of microliter injection volume was recently reported (Trimzi et al., 2019). The analysis demonstrated the dependency of the injection dynamics on parameters such as impulse force, nozzle diameter, and length. The idea of this study was that multiple layers of piezoelectric actuator would instantly expand and move the fluid through a fine nozzle, avoiding the use of a needle injector. Also, as the applied voltage determined the expansion of the piezoelectric layers, this experimental variable could be used to electronically control the injection volume and directly affected the jet formation phenomenon.

Furthermore, some *in vitro* research studies on needle-free jet injection dynamics were recently published (Rohilla and Marston, 2019; Zeng et al., 2019). These analyzed the influence of nozzle orifice diameter, of the injection volume, of the liquid viscosity, of tissue stiffness and of the standoff distance between the nozzle orifice and the skin surface on the jet injection dispersion pattern and penetration depth in the tissue. Width and length diffusion region would be influenced by the ejection volume and the orifice diameter, respectively. Conspicuously, one of these studies showed that fluid penetration depth was increased by increasing the standoff distance between the nozzle orifice and a gel surface, which could dependent on the model surface used.

Among *in vivo* studies, needle-free drug delivery systems have been recently used to deliver vaccines to the sublingual and buccal tissue of rhesus macaques by using a commercially available needle-less injector and, in this way, to test the viability as an effective and practical route of administration to induce immunity against HIV-1 (Jones et al., 2019). In addition, needle-free injectors have been tested in Chinese patients with type 2 diabetes (Xing et al., 2019). In this trial, insulin glargine was administered by another commercially available needle-free injector for 1–2 weeks. Interestingly, patients who received the needle-free administration demonstrated less discomfort, fear and pain than patients receiving conventional needle-based injections. Also, the insulin dose required for glycemic control was reduced by using the needle-free system and it was a positively correlated with the degree of that dose reduction. This study determined that needle-free injection system would reduce the adverse effects of high insulin doses, topical reactions and fear of injections, which should help improving patient compliance to this treatment.

In addition to reduce pain and stress, this technology avoids needle disposal, enables self-administration and has been show to offer consistent delivery of vaccines and other medications. Its main drawback is the remaining moisture of the skin after administration, which can, if not taken care of, harbor dust and other adverse impurities.

## Discussion

During the past few years an interesting number of new micro-electro-mechanical devices for controlled drug release applications have emerged. Indeed, the unique physical and analytical functions together with electrical components and aseptically manufacture suggest that they have important applications in medicine, especially as therapeutic agents delivery systems. At the forefront of research in this area is the development of actuation mechanisms to deliver the pharmaceutically cargo in controlled and exact doses. Moreover, considerable effort has been devoted to the design and fabrication of these devises, including single or multiple reservoirs, implantable or transdermal devices and to marry these novel micro-electro-mechanical devices with the sophisticated characteristics of biological systems, in order to achieve an effective delivery of therapeutic agents *in vivo*.

The different studies presented in this review highlight the potentialities of these devices with different engineering designs and actuator mechanisms and that the application of MEMS devices for controlled drug release is an emerging field with important implications in human healthcare. Table 3 summarizes the advantages, disadvantages and final applications of the implantable and transdermal described MEMS devices. Some of them showed prolonged and/or controlled release profiles of therapeutic compounds such chemotherapeutic drugs and hormones but only reached the *in vitro* stage needing further research. Others, especially those based on multi-reservoir design, were tested *in vivo* and a reduced number of them have been tested in human clinic trials. In this sense, it is important to note that these devices are especially desirable and useful in the case of providing release of high potent drugs which evoke a given response at very low concentrations. That offers the possibility to load higher individual doses of a given drug in ultra-minimally invasive devices and to release it for longer periods of time. Alongside, biodegradable polymeric microneedles and needle-free injection technology allow painless and cost-effective transdermal drug administration, increasing patient compliance. Although many advances have been made on device miniaturization, biocompatibility, remote control, operating autonomy, low energy consumption, programming of varied release profiles, controlled and/or pulsatile release for prolonged periods of time, there is still no such versatile device to date that can gather all these interesting properties. In view of the traveled path, it is envisioned that further investigations and technological developments would make this a reality in a short time.

**TABLE 3:** Advantages, disadvantages, and final applications of implantable and transdermal MEMS devices for drug delivery.

Route of administration	Working principle	Approximate device dimensions	Advantages	Disadvantages	Final applications	References
Implantable	Piezoelectric-based micropumps	(0.70 × 0.13) mm	High actuation forces, fast response times, and precise volumetric dosing of aqueous fluids.	Complex manufacture, special care when processing and mounting PZT discs, high actuation voltages required.	Pending	
	Magnetic-based micropumps	(6 Ø × 0.59) mm	Battery-less operation and enhanced miniaturization, large actuation force.	Potential harmful effects of magnetic field application (especially in chronic treatments)	Docetaxel delivery for diabetic retinopathy.	Pirmoradi et al., 2011b; Zachkani et al., 2015
	Phase-change based micropumps	(8 mm × 8 mm × 3 mm)	Blocked flow in the off-state, drug reservoir isolated from the delivery target without consuming energy, low actuation voltage, wireless operation.	Slow response time (especially during cooling), low frequency of operation and limited materials.	Pending	
	Electrochemical-based micropumps	10 mm × 10 mm × 2 mm	Simple fabrication, easy integration with microfluidic systems, continuous and smooth drug delivery, large mechanical displacement with low power consumption, battery-less operation when using TENG.	Generated bubbles can collapse into water causing an unsteady and unreliable drug release.	Phenylephrine delivery for blood pressure increase. siRNA-gold nanoplex-based drug for anti-tumoral therapy.	[Bibr B75][Bibr B36]
	Acoustic-based micropumps	75 mm × 50 mm × 1 mm	High precision, small volume, programmable capability, low cost.	Size of bubbles could be affected upon time resulting in changes in the resonant frequency of the bubbles.	Pending	
	Electrochemical dissolution of capping membranes	N/S	Especially useful for potent drugs, programmable capability to achieve diverse release profiles including pulsatile delivery.	Potential harmful effects of soluble gold chloride complexes formed upon dissolution of reservoir capping membranes, limited reservoir volumes.	Carmustine (BCNU) delivery for brain tumors treatment.	Li et al., 2005
	Electrothermal aperture of capping membranes	(13 mm × 5.4 mm × 0.5 mm)	Especially useful for potent drugs, programmable capability to achieve diverse release profiles including pulsatile delivery, wireless operation, multi-drug release capability.	Limited reservoir volumes, need for battery increase device dimension.	Leuprolide delivery for prostate cancer and endometriosis treatments. Human parathyroid hormone fragment delivery for osteoporosis treatment.	[Bibr B100][Bibr B29]
	NIR-based aperture of capping membranes	27 mm × 11.5 mm × 9.5 mm	Minimally invasive, battery-less operation, pulsatile release capability.	Potential lack of patient compliance due to discomfort, need for a light source localized outside the body.	Human growth hormone for deficiency treatment.	Lee et al., 2019
	Passive release	(0.82 Ø × 3) mm	Simple fabrication, multi-drug release capability.	Limited release profiles	Chemotherapeutic drugs to test tumor *in vivo* sensitivity.	Jonas et al., 2015
Transdermal	Silicon/carbon microneedle-based devices	100–200 mm^2^	Painless, ease of self-administration	Complex fabrication, not biodegradable materials, safety issues raised	Insulin delivery for diabetes treatment. Plasmid DNA encoding hepatitis B surface antigen for immune response induction.	[Bibr B82][Bibr B86]
	Polymeric microneedle-based devices	100 mm^2^	High efficiency, biodegradable materials, cost-effectiveness, ease of self-administration, painless.	Not precise and uniform large-scale manufacturing	Sustained release of contraceptive drugs. Prolonged release of doxorubicin for cancer treatment.	[Bibr B69][Bibr B77]
					Glucose-responsive insulin delivery.	Chen et al., 2019
					STAT3 siRNA delivery for the treatment of melanoma.	Pan et al., 2018
					Rapid release of antigen for immune response induction.	He Y. et al., 2018
	Needle-free injectors	Comparable to a 25-guage needle	Painless and stress-less, no need for needle disposal, self-administration capability.	Remaining moisture of skin after administration may harbor dust and other adverse impurities.	Induce immunization against HIV-1.	Jones et al., 2019
					Insulin glargine delivery for glycemic control.	Xing et al., 2019

*N/S, not specified; TENG, triboelectric nanogenerator; NIR: near-infrared radiation; Ø: diameter.*

## Author Contributions

LV and NS carried out the bibliographic search, analyzed and discussed the manuscript in the field, prepared the figures and tables, and helped with manuscript writing. MB helped with the manuscript writing, discussion, and revision. MD and PC organized the manuscript and participated in its discussion, writing, and editing. All authors contributed to the article and approved the submitted version.

## Conflict of Interest

The authors declare that the research was conducted in the absence of any commercial or financial relationships that could be construed as a potential conflict of interest.
